# Cultivating special education teacher well-being: Nurturing connection in professional learning communities

**DOI:** 10.4102/ajod.v14i0.1547

**Published:** 2025-08-05

**Authors:** Madira Matjeni, Sarina de Jager

**Affiliations:** 1 Department of Humanities Education, Faculty of Education, University of Pretoria, Pretoria, South Africa

**Keywords:** professional learning communities, teacher well-being, connection, special education teacher, autism spectrum disorders

## Abstract

**Background:**

Special education teachers, especially those working with learners with autism spectrum disorders (ASD), often face significant stress and burnout, affecting their well-being. This study explores teacher well-being in special education, motivated by personal experiences as a novice teacher in a special educational needs school.

**Objectives:**

The study aimed to provide special education teachers with an opportunity to collaboratively examine and address well-being challenges and opportunities within a professional learning community, focusing on belonging, competence, and autonomy in Learners with Special Educational Needs (LSEN) settings. Utilising a phenomenological research design, the study involved eight teachers working with learners with ASD.

**Method:**

Data collection methods included observations, semi-structured interviews, and focus group discussions to comprehensively explore their lived experiences. The findings highlighted the profound impact of challenging experiences on teacher well-being and the lack of sufficient support structures. Participants emphasised the critical role of social support in enhancing teacher well-being.

**Results:**

Participants defined well-being as emotional, physical, and holistic, including social and family aspects. While working with learners with ASD was meaningful, it also caused significant stress due to their complex needs. Daily challenges shaped teachers’ professional identity and competence. Connections with colleagues were valued, but a lack of institutional support and a restrictive school culture hindered well-being. When given autonomy, participants creatively co-developed well-being interventions, though support from management was essential for implementation.

**Conclusion:**

This research highlights the essential role of fostering a sense of belonging and connection within professional learning communities for special education teachers. It calls for increased accountability in developing well-being support structures and comprehensive training programmes tailored to the needs of novice educators in special education. By nurturing connections, fostering competence, and ensuring autonomy, the well-being of special education teachers, particularly those working with learners on the autism spectrum, can be significantly improved.

**Contribution:**

This study contributes to enhancing well-being support structures and training programmes for special education professionals.

## Introduction

The extensive body of research on teacher stress and burnout over the past two decades highlights the growing concern regarding educators’ well-being (Bottiani et al. 2019; Cancio et al. 2018; Zhang, Zhang & Hua 2019). Teaching is widely recognised as a high-stress profession and teachers in special education face additional challenges because of the complexity of working with students with disabilities. Special educational needs educators often report emotional exhaustion, depersonalisation, and reduced personal accomplishment, leading to increased turnover rates and a diminished sense of professional identity (Botha, De Jager & Evans 2023). Internationally, studies have examined stress in teaching alongside other high-pressure professions such as nursing, policing, and firefighting. Many teachers struggle to maintain their commitment, sometimes losing their sense of purpose (Granevald, Vinterek & Strömsten 2024). In contrast, research on teacher stress and burnout in South Africa and the broader Global South is still developing. Special education teachers in South Africa experience significant work-related stress because of inadequate resources, overcrowded classrooms, and a lack of institutional support (Murangi, Rothmann & Nel 2022). Sessions et al. (2017) found that limited career progression and low salaries further exacerbate stress levels, contributing to high rates of attrition. Multiple factors contribute to poor teacher well-being, particularly in special education. Studies highlight the emotional and physical demands of working with children with disabilities, who often require significant support in daily routines, emotional regulation, and learning (Chen et al. 2023). Teachers working with students diagnosed with autism spectrum disorder (ASD) face additional challenges, including behavioural management difficulties, increased workload, and lower self-efficacy (Love et al. 2019). Research also indicates that teachers in special education schools frequently experience frustration, isolation, and depression, leading to burnout and high turnover rates (Fu et al. 2022). Teacher stress negatively affects job satisfaction, health, and retention rates. Studies indicate that many special education teachers leave the profession within the first 5 years, with at least 50% resigning because of burnout (Fu et al. 2022). The impact of teacher attrition is evident in schools, where high turnover rates disrupt learning environments and affect student outcomes. In addition, teachers experiencing compromised well-being struggle to bring creativity into the classroom, ultimately harming learner engagement (Turner 2016). Recognising the critical role of teacher well-being, international and local efforts have been made to develop support systems for educators. The Job Demands-Resources (JD-R) model suggests that increasing job resources – such as administrative support, professional development, and peer collaboration – can mitigate stress (Bakker & Demerouti 2017). South Africa’s policy framework, including the Bill of Rights and Education White Paper 6 (Department of Education 2001), has attempted to promote inclusive education and support for special needs educators. However, challenges remain in implementing effective community-based interventions and mental health support programmes Sessions et al. (2017).

Over the past decade, awareness and diagnosis of ASD have increased, partly because of enhanced diagnostic criteria and global access to medical knowledge (Solmi et al. 2022). Despite growing awareness, public understanding of ASD remains insufficient, and even healthcare professionals often lack adequate training (Davin 2020; Hayat et al. 2019). This knowledge gap affects educators, who frequently navigate the complexities of ASD with limited resources and training, further contributing to stress and burnout. Teacher well-being is a critical issue that directly impacts educational outcomes. While international research has extensively examined teacher stress and burnout, studies focusing on special needs educators in South Africa and the Global South remain limited. The existing literature highlights significant job demands, emotional exhaustion, and high attrition rates among special education teachers.

## Research methods and design

This study employed a qualitative phenomenological design to explore the subjective well-being of teachers working with learners diagnosed with ASD within a professional learning community (PLC). A PLC is a group of educators who collaborate to improve teaching and student outcomes through continuous learning and shared practices, through ongoing reflective dialogue, shared leadership, and continuous professional development (DuFour 2004). The PLC was used in the study as a platform for teachers to engage in discussions about their well-being and their experiences in teaching learners with ASD. The PLC provided teachers with a safe space for emotional support, professional reflection, and shared learning. It fostered collaboration, trust, and collective problem-solving, helping participants normalise their challenges and develop strategies for coping with stress. Data collection spanned from April to July 2022 and involved three primary methods: focus group discussions, semi-structured interviews, and observations. The use of multiple data sources ensured triangulation, enhancing the study’s credibility.

Four focus group discussions were conducted over a 2-month period at a Learners with Special Educational Needs (LSEN) school in Pretoria, South Africa. Each session was scheduled after school hours to accommodate participants’ availability. The focus groups involved eight teachers, including both novice and experienced educators, with the researcher participating as an active participant observer. Sessions took place in a classroom at the school and were guided by a structured protocol aligned with the research questions. The discussions were designed to foster dynamic engagement on teachers’ subjective well-being and their experiences within the PLC. The discussions focused on the following key areas: teachers’ experiences of working with learners with ASD, institutional efforts to support teacher well-being, the impact of teaching on personal and professional well-being, and experiences and perceptions of PLC participation. Each session lasted between 23 and 56 min. The researcher facilitated and moderated the discussions, ensuring that all voices were heard. Observations were recorded on participant engagement, body language, tone, and group dynamics to capture non-verbal cues that complemented the verbal data. All sessions were audio-recorded and transcribed verbatim for analysis.

Three semi-structured interviews were conducted with selected participants who had either missed a focus group session or had provided insights that required further elaboration. Each interview was scheduled for after school hours, lasting approximately 45 min. The interviews followed a flexible format, allowing for open-ended responses while maintaining a structured flow based on pre-determined questions. The interview questions were guided by the following research objectives: how can a PLC enhance the well-being of special education needs (SEN) teachers, what challenges and support structures exist for teacher well-being, and how do SEN teachers perceive their role in promoting well-being? Additional follow-up questions were posed as needed to clarify or expand upon points raised in the focus group discussions. The interviews were audio-recorded with participants’ consent, transcribed, and cross-checked against recordings to ensure accuracy.

Observations were integrated as a third data collection method to provide contextual depth to the focus group and interview data. These took place during all four focus group discussions, with the researcher assuming the role of a participant observer. Observational data focused on participants’ level of engagement and participation, interpersonal interactions and collaboration within the PLC, and non-verbal cues such as facial expressions, gestures, and body language. Field notes were taken during and immediately after each session to ensure accuracy. While observational data contributed to the broader understanding of teacher well-being, it was primarily used for triangulation and was not separately reported in the findings.

Given that the researcher was also a teacher at the research site, careful measures were taken to mitigate potential coercion. Participation was entirely voluntary and all participants were explicitly informed that their decision to take part or withdraw at any time would have no impact on their professional standing. To further ensure openness, clear communication emphasised that there were no right or wrong responses, and participants were encouraged to share their experiences freely. Confidentiality was strictly maintained and all data were anonymised to prevent any identification of individual responses. These steps helped establish an environment where participants felt comfortable expressing themselves without concern for undue influence.

### Participants

The study included seven teachers from an SEN school in Pretoria, South Africa, all of whom taught in the ASD phase. Participants were purposefully selected based on their experience in teaching learners with ASD. Their qualifications ranged from postgraduate certificates in education to bachelor of education degrees, with specialisations in foundation phase, early childhood development, intermediate and senior phase, and further education and training phase. Teaching experience varied, with participants having between 1 and 10 years in general teaching, while experience specifically in the ASD phase ranged from less than a year to 5 years. The study aimed to capture the lived experiences of both novice and experienced teachers, ensuring a diverse perspective on well-being within this specialised teaching context.

### Data analysis

Interpretative phenomenological analysis was employed during this study as a data analysis tool, allowing us to explore the participants’ personal experiences in depth (Smith, Flowers & Larkin 2009). One of the key features of interpretative phenomenological analysis is that it enables researchers to gain an understanding of how participants make sense of their experiences. This was achieved using detailed, open-ended interviews, which were then analysed using a rigorous, step-by-step process:

**Organisation:** Focus group discussions, in-depth interviews, and observations were used to collect data with the participants. We then transcribed the audio-recorded interviews and focus group discussions and analysed them in Microsoft Word. This allowed for the organisation of the data into smaller pieces, which made it easier to interpret and understand.

**Perusal:** We examined the collected data several times to understand what the data contained as a whole. We read and reread the data, ensuring accuracy between the audio-recorded interviews and focus group discussions and the transcripts. This process is known as data reduction, and it is an important step in data analysis.

**Classification:** We classified the data by dividing them into themes and sub-themes. Themes refer to broad categories that provide a general overview of the subject matter, and sub-themes refer to narrower categories that offer more specific information about the theme.

**Synthesis:** We searched for connections across themes to create a more comprehensive understanding of the topic, aiding in creating a well-rounded argument.

**Induction:** We adopted a qualitative approach informed by both existing literature and empirical data. As researchers, we first reviewed relevant literature to frame our understanding of the topic, followed by the collection of primary data through interviews and focus group discussions to explore participants’ perspectives in depth. The data were then analysed using interpretative phenomenological analysis (IPA), which enabled us to examine how participants made sense of their experiences while allowing themes to emerge organically from the data.After thoroughly investigating the primary and secondary sources of information, we developed our findings, conclusions, and recommendations.

### Ethical considerations

Ethical approval for this study was granted by the Research Ethics Committee of the Faculty of Education at the University of Pretoria (EDU152/21). The research adhered to the three core ethical principles outlined in The Belmont Report: beneficence, respect for persons, and justice. These principles guided the study to ensure ethical integrity throughout the research process. Beneficence was upheld by prioritising the well-being and safety of participants. Measures were taken to minimise potential risks and protect their rights. Respect for individuals was maintained through informed consent and the safeguarding of vulnerable groups. Given that the study took place in a school setting with students with disabilities, additional precautions were implemented to protect participants. Pseudonyms were assigned to participants, teachers, and the school itself to ensure anonymity. An asterisk (*) was placed after pseudonyms to indicate that they were not the individuals’ real names. Justice was ensured by maintaining fairness in the distribution of benefits and burdens among participants. Permissions were obtained from relevant stakeholders, and key ethical considerations – including informed consent, voluntary participation, anonymity, and confidentiality – were thoroughly explained and implemented. Given the involvement of a school for learners with disabilities, extra care was taken to protect participants and uphold data accuracy and integrity.

The application of ethical principles, participant protection, and data integrity remained a priority throughout the study, ensuring ethical rigour in research involving vulnerable populations.

## Results

This section presents key insights derived from focus groups and semi-structured interviews, employing thematic analysis to identify core themes. The findings explore participants’ conceptualisation of well-being, meaningful teaching experiences, challenges hindering well-being, and available support mechanisms.

### Teachers’ perceptions of well-being

Participants described well-being as a multifaceted concept encompassing emotional, physical, and social dimensions. Michael* explained, ‘It’s emotional and physical, like how you’re feeling … your body… and also emotional, on the inside’, while Athambile* emphasised inner peace, stating, ‘Well-being has to do with your inner being … your emotion, of course … your inner peace’. Onu* suggested a holistic perspective, asserting, ‘It must be a holistic thing. The whole of you must be okay’. These reflections align with research on job satisfaction and emotional resilience (Deci & Ryan 2012; Mertler 2016), reinforcing the need for supportive professional environments.

### Meaningful experiences and well-being

Participants highlighted how positive teaching experiences contributed to their well-being, particularly through a sense of purpose and emotional connection with learners. Anele* stated, ‘It gives you a sense of self-worth; you’re also contributing to your household’, while Jessica* noted the stability employment provided, saying, ‘Wow, I got a job! … I’m gonna get money monthly!’ Small achievements among learners were deeply fulfilling, as Leo* described, ‘With each little achievement these learners go through, for you, it’s a big hoo-ha’. The reciprocal nature of well-being was evident, where participants found emotional sustenance through meaningful teacher-student interactions. These insights align with Basic Psychological Needs Theory, which emphasises autonomy, competence, and relatedness as key motivators in professional fulfilment (Ryan & Deci 2000).

### Challenges: Emotional and physical exhaustion

Despite these positive experiences, teachers faced significant emotional and physical exhaustion. Margaret* recalled, ‘Back in 2018 … a child had a meltdown, and he fractured my carpals… they’ve attacked me with plastic knives … it just happens so quick’. Michael* described the prolonged stress, ‘I’ve been sicker … constantly stressing about things the children do … I have to be aware of’. Participants also noticed emotional exhaustion because of deep attachments with learners. Leo* shared, ‘You tend to get too attached… you know each learner and their socio-economic background’. These findings align with previous research on compassion fatigue and burnout in special education (Fu et al., 2022; Lee, Lee & Jang, 2021), highlighting the urgent need for structured mental health support and stress management training.

### School culture and institutional support

A prevalent theme was the disconnect between formal well-being initiatives and their practical implementation. Participants described a culture of ‘window-dressing’, where surface-level efforts masked deeper structural issues. Anele* noted, ‘By the time they come to our schools, the Principal and HoDs always take them to the nicest classes and the nicest teacher’, while Leo* added, ‘And the well-behaved learners’. Although a wellness committee existed, it was perceived as ineffective. Onu* stated, ‘There is a Wellness Committee, but… they are not featuring … for the well-being of teachers’. This mirrors broader concerns in educational research that emphasise the disparity between policy and practice in well-being frameworks (Naidoo 2019; Park & Shin 2020).

### Teacher identity, competency, and imposter syndrome

Many participants struggled with imposter syndrome, questioning their professional competence. Leo* admitted, ‘You tend to also second guess yourself’, while Jessica* noted, ‘I’m very anxious … about my personal growth’. The lack of sufficient professional development opportunities further exacerbated self-doubt. This aligns with literature on imposter syndrome, which identifies self-perceived inadequacy as a key stressor among educators (Huecker et al. 2022). Mentorship and structured peer support within PLCs emerged as potential interventions to mitigate these concerns.

### Professional learning communities as a support mechanism

Professional learning communities played a pivotal role in fostering emotional resilience and professional growth. Participants valued the shared experiences, with Margaret* describing it as ‘a safe space’ to express concerns without fear of judgement. The PLC encouraged self-reflection, with Onu* realising, ‘You need to look after yourself to be able to look after others’. Jessica* highlighted the transformative potential of PLCs, stating, ‘Growth is not just academic; it’s also about inner peace and self-care’. These insights align with broader research advocating for PLCs as essential tools for sustaining teacher engagement and professional resilience (Molina & Lopez 2019).

### Facilitating teacher well-being

Participants proposed various strategies to enhance well-being, including recognition from school leadership, improved staffroom environments, and dedicated funds for well-being initiatives. Jessica* suggested a peer support group, while Michael* recommended creating a ‘chill corner’ for teachers to decompress. Anele* emphasised the need for budget allocation, stating, ‘If we can maybe put in the extra amount of budget … I think it would go forward’. These recommendations align with research emphasising the role of institutional recognition, team-building, and support systems in enhancing teacher well-being (Brodie 2021; Gorman 2019).

This study highlights the complexities of teacher well-being within special education, emphasising the interplay between meaningful experiences, emotional challenges, and institutional support. While PLC emerged as a crucial support structure, systemic interventions – such as leadership accountability, structured mentorship, and dedicated mental health resources – are essential for long-term sustainability. Addressing these factors holistically can create a more supportive and fulfilling work environment for educators, ultimately benefiting both teachers and learners.

## Recommendations

The findings revealed the need for SEN education teachers who experienced limited opportunities to connect with colleagues to engage in critical conversations. Therefore, we recommend opportunities for SEN teachers to engage in critical conversations to explore the various issues that teachers face in this field. Critical conversations open dialogue for teachers, offering different perspectives on an issue and allowing expansion and growth (Harste 2000). The core values of critical conversations are engagement, learning opportunities, and resolving issues within a community. Critical conversations should take place within PLCs, opening paths for reflection on the part of teachers (Cox et al. 2018). Reflections alone do not suffice in these discussions; they also need to be critical, requiring teachers to be mindful or ‘critically conscious’. These discussions provide teachers with new ways of thinking about old problems and allow for innovative solutions (Vetter, Schieble & Martin 2021; Zúñiga, Lopez & Ford 2012). They also help build stronger relationships with colleagues.

The data revealed that well-being support structures were in place but underutilised. This is concerning as it suggests that schools are not effectively measuring or taking accountability for teacher well-being. The study recommends that LSEN schools monitor the effectiveness of support structures to track teacher retention rates. If retention rates increase after supports are put in place, then it is likely that the supports are adequate. Another way to measure the effectiveness of support structures is to survey teachers before and after support is put in place. If there is an increase in satisfaction levels, the supports are likely adequate. Another recommendation is for the school management team to acknowledge the emotional needs of SEN teachers and support them adequately by making provision for professional support through the school’s budget. Support from school management and the School Governing Body (SGB) is key to retaining well-trained, talented teachers. The study recommends training opportunities and team-building activities to foster connections among SEN teachers. This will help teachers feel more supported and appreciated for their valuable contributions. In addition, giving SEN teachers more autonomy will help improve job satisfaction levels. There is a lack of training for teachers who work with students with disabilities at all levels of education and teachers with special needs have little or no training in teaching and coping with learners with disabilities. We therefore recommend that the higher education sector aim to improve this gap, which results from burnout, stress, and a lack of experience, as it leads to detrimental health issues. The lack of training in this field fails SEN teachers and learners. For this reason, we further recommend that all undergraduate teaching qualifications include modules based on learners with disabilities to open pathways for teachers, giving them future opportunities to work with learners with special needs.

## Conclusion

This study underscores the critical role of SEN teacher well-being in shaping the overall health of schools and the quality of education provided to learners. The findings highlight both the meaningful aspects of teaching learners with ASD and the substantial challenges, including emotional and physical exhaustion, inadequate institutional support, and impostor syndrome. While PLCs offer a valuable space for emotional support, collaboration, and professional growth, systemic barriers continue to impact teacher retention and job satisfaction. Given that SEN teachers are particularly vulnerable to high levels of stress and burnout, it is imperative to establish and maintain strong support structures that prioritise their well-being. A unique contribution of this study lies in its application of PLCs as tools to foster teacher well-being, offering a collective approach to stress management and professional resilience. Ensuring teacher well-being has far-reaching implications. When teachers are happy and healthy, they are more effective in the classroom, fostering better learning outcomes. Furthermore, well-supported teachers are more likely to remain in the profession, reducing turnover and promoting stability for both learners and schools. Investing in teacher well-being can also attract and retain skilled professionals in special education, addressing the ongoing shortage of trained educators. In addition, creating a culture of support for teachers generates a positive ripple effect throughout the school community, enhancing engagement, collaboration, and overall morale.

The study’s strengths lie in its qualitative approach, which captures the lived experiences of teachers and provides insight into the role of PLCs in fostering well-being. However, limitations include the small sample size and the focus on a single school, which may limit broader applicability. Moving forward, targeted professional development in special education, institutional accountability, and the integration of mental health support into school policies are necessary steps to sustain teacher well-being. Future research should explore the long-term impact of PLCs on teacher retention and job satisfaction across diverse educational settings. By prioritising teacher well-being at both school and policy levels, sustainable improvements in special education can be achieved, ultimately benefiting both educators and learners.
